# *In Vivo* Biocompatibility of PLGA-Polyhexylthiophene Nanofiber Scaffolds in a Rat Model

**DOI:** 10.1155/2013/390518

**Published:** 2013-07-23

**Authors:** Anuradha Subramanian, Uma Maheswari Krishnan, Swaminathan Sethuraman

**Affiliations:** Center for Nanotechnology & Advanced Biomaterials, School of Chemical & Biotechnology, SASTRA University, Thanjavur 613 401, Tamil Nadu, India

## Abstract

Electroactive polymers have applications in tissue engineering as a physical template for cell adhesion and carry electrical signals to improve tissue regeneration. Present study demonstrated the biocompatibility and biodegradability of poly(lactide-*co*-glycolide)-poly(3-hexylthiophene) (PLGA-PHT) blend electrospun scaffolds in a subcutaneous rat model. The biocompatibility of PLGA-undoped PHT, PLGA-doped PHT, and aligned PLGA-doped PHT nanofibers was evaluated and compared with random PLGA fibers. The animals were sacrificed at 2, 4, and 8 weeks; the surrounding tissue along with the implant was removed to evaluate biocompatibility and biodegradability by histologic analysis and GPC, respectively. Histology results demonstrated that all scaffolds except PLGA-undoped PHT showed decrease in inflammation over time. It was observed that the aligned PLGA-doped PHT fibers elicited moderate response at 2 weeks, which further reduced to a mild response over time with well-organized tissue structure and collagen deposition. The degradation of aligned nanofibers was found to be very slow when compared to random fibers. Further, there was no reduction in the molecular weight of undoped form of PHT throughout the study. These experiments revealed the biocompatibility and biodegradability of PLGA-PHT nanofibers that potentiate it to be used as a biomaterial for various applications.

## 1. Introduction

Conjugated polymers such as polyaniline (PANi), polypyrrole (Ppy), and poly(3,4-ethylenedioxythiophene) (PEDOT) offers greater advantage for the development of tissue engineering scaffold especially for neural regeneration due to its electrical conductivity [1–4]. Such polymers exhibit completely different electronic configuration than insulating polymers by having alternating single and double bonds [5]. Further, incorporation of dopants or impurities into the conjugated polymers can improve the conductivity by creating charge carriers into the polymer backbone [6]. However, apart from electrical conductivity, biocompatibility and biodegradability are the two main ideal properties required for biomaterials [7]. These biomaterials should not elicit any short- or long-term immune response. Similarly, polymers and its degradation products should not be toxic to cells or tissues or affect the normal physiological functions [8]. Consequently, biomaterials can either elicit inflammation or exhibit no response in the tissues [7]. Implantation of inert biomaterials often causes fibrous tissue encapsulation, whereas toxic biomaterials lead to cell death.

In recent times, several research groups have tested different conducting polymers in terms of cell viability and tissue response toward the materials using animal models, thereby demonstrating its biocompatibility from fair to good [9]. Conversely, certain polymers such as polyethylene induced tumor formation after implanting into the subcutaneous rat model [10]. Polypyrrole (Ppy) has been found to have no significant long-term inflammatory response after six weeks of subcutaneous implantation in rat model [11]. However, Ppy is rigid, brittle, and is not degradable because of its highly conjugated molecular backbone [1]. Similarly, emeraldine salt as well as base form of polyaniline (conducting and nonconducting) did not elicit any immune responses in rodents [12, 13]. Additionally, different research groups have evaluated the cytotoxicity of polyaniline *in vitro *using various cell types such as fibroblast, pheochromocytoma (PC12), and cardiac myoblast (H9c2) cells [14, 15]. Bidez III et al. demonstrated that cell adhesion and proliferation on PANi was comparable to tissue culture polystyrene [16]. Like polyaniline and polypyrrole, poly(3,4-ethylenedioxythiophene) (PEDOT) has been extensively investigated for tissue engineering applications because of its unusual environmental and electrochemical stability in oxidized state [17]. Polythiophene derivatives have been used to develop molecular actuators to mimic natural muscles [18]. Subcutaneous implantation of PEDOT in mice model demonstrated the absence of inflammatory response after one week and the formation of a thin layer of tissue capsule after 28 days [19]. Moreover, pure highly regioregular poly(alkylthiophene) has proven to form biocompatible self-assembled monolayers [20].

Though there are various kinds of scaffolds available for neural regeneration, electrospun nanofibers offer immense promise for the functional restoration of the nerve tissue. Properties such as high surface-to-volume ratio, controllable pore size with porosity, and oriented topography with the native ECM architecture can direct the neural cells towards the distal end of the denuded nerve end with the good contact guidance [21]. However, organic solvent solubility is the key limiting factor for this technique since most conducting polymers such as polypyrrole, polyaniline, and polythiophenes are insoluble or very sparingly soluble in organic solvents due to their rigid polymer backbone [22, 23]. This intractability problem can be overcome by blending with some conventional polymers. 

Recently, a novel blend of PHT-PLGA electrospun scaffolds has proven to be promising substrate for neural regeneration by improving Schwann cell proliferation, controlled degradation with good electrical conductivity [24]. To the best of our knowledge, there are no reports available on *in vivo* biocompatibility as well as biodegradability. Hence, in the present study, we have chosen to fabricate the blend scaffold of poly(3-hexylthiophene) and poly(lactide-co-glycolide) (PHT-PLGA) by mixing the two polymers in order to achieve the amalgamated properties of both polymers. PHT is chosen for its good organic solvent solubility, for the ease of processing to form uniform nanofibers, outstanding environment stability with structural versatility, and electrical properties to establish electrical cue for controlling cell fate, while PLGA is preferred for its recognized biodegradable and biocompatible properties [22, 24]. In addition, PHT is p-doped using sulfonic acid which improves the electrical property of the polymer [24, 25].

The main objective of this study was to determine the *in vivo* tissue responses and degradability of random and axially aligned PLGA-PHT blend nanofibers and also to evaluate the effect of doping on both tissue response and biodegradation, since the extent of foreign body reaction depends on size, topography, degradation, and composition of the implant [26]. The performances of electrospun scaffolds in rat model assessed were random PLGA-undoped PHT, random PLGA-doped PHT, and aligned PLGA-doped PHT nanofibers and compared to control (PLGA random nanofibers).

## 2. Materials and Methods

### 2.1. Materials

PLGA (Mw 118 KDa) was purchased from Lakeshore Biomaterials, Birmingham, AL, USA. PHT was obtained from Rieke Metals, Inc. Lincoln, NE, USA, and (+/−) 10-Camphor sulphonic acid was purchased from Sigma Aldrich Chemicals, Bangalore, India. Dichloromethane (DCM) and N,N-dimethyl formamide (DMF), chloroform (CHCl_3_) were purchased from Merck, India, and were used without any further purification.

### 2.2. Fabrication of Random and Aligned Nanofibrous Scaffolds

Doped polyhexylthiophene was prepared by dropwise addition of dopant camphorsulfonic acid in chloroform into PHT solution and stirred overnight. The solvent was completely evaporated using rotary vacuum evaporator (Rotavapor R215, Buchi, Switzerland). This powder was used to prepare PLGA-doped PHT blend solution. The random PLGA, PLGA-undoped PHT, and PLGA-doped PHT nanofibers and axially aligned PLGA-doped PHT nanofibers were developed through electrospinning under optimum conditions [24]. Briefly, 12% (w/v) of PLGA in a mixture of dichloromethane (DCM) and N,N-dimethyl formamide (DMF) (9 : 1) was prepared. PLGA-undoped PHT blend solution was prepared by dissolving 10% (w/v) of PLGA and 10% (w/v) undoped-PHT in a mixture of chloroform and N,N-dimethyl formamide (DMF) (9 : 1). Similarly, 10% (w/v) of PLGA and 10% (w/v) of doped PHT were blended in chloroform and DMF in 9 : 1 ratio. These solutions were fed into a 5 mL glass syringe, the flow rate was controlled by syringe pump (KD Scientific 200, USA), and the high-voltage was applied using a high voltage power supply (Zeonics, India). The random nanofiber sheets were collected on aluminium foil fixed onto a grounded metal collector. The axially aligned nanofibers of tubular scaffolds were collected using a grounded rotating mandrel (3 mm in diameter) with small insulating gaps [21]. The speed of the mandrel was controlled using a motor (Remi, India) to align the fibers in the longitudinal direction. The random nanofiber mats were cut into small circular discs of about 10 mm in diameter, and aligned nanofiber tubes with 10 mm length were taken for further characterization. Before implantation, each side of implants was sterilized under ultraviolet (UV) light for 30 minutes.

### 2.3. Animal Model and Implantation

Thirty-six male Wistar rats (*Rattus norvegicus*), each weighing 200–250 g, were used for animal experiments. Animals were randomly divided into four groups of nine rats each. All rats were kept in an individual cage and were housed in a temperature-controlled facility. The surgical procedures were approved by Institution Animal Ethics Committee of SASTRA University. Every group (9 rats) was assigned randomly to three different time points (2, 4, and 8 weeks) with 3 rats of each. Intraperitoneal injections of Ketamine (30 mg/kg body weight) and Xylazine (13 mg/kg body weight) were administrated to anesthetize the animals. The dorsal area of the animals was shaved and sterilized with 70% ethanol solution. Using a sterile surgical blade no. 22 (Magna Marketing, India), an incision of about 12 mm was made on the dorsum of animals. A subcutaneous pouch was created on both sides of the incision, and an implant was inserted into each pocket. Upon implantation of the polymer into the pouch, the cut was sutured using a nonabsorbable surgical black braided silk thread (Relyonpac, India). Animals in Groups I, II, III, and IV were implanted with random PLGA, PLGA-undoped PHT, PLGA-doped PHT, and aligned PLGA-doped PHT nanofibers, respectively. The sutures were removed 7 days after surgery.

### 2.4. Histology Studies

At the end of each time point (2, 4, and 8 weeks), rats were euthanized using an overdose of pentobarbital (75 mg/kg) followed by carbon dioxide asphyxiation. The implant and the tissues surrounding the implant were excised. The tissues surrounding the implant were fixed in 10% formalin solution for 7 days. Before embedding in paraffin wax, the tissue samples were dehydrated in an Automatic Tissue Processor (Yorco YSI103LT, Yorco Scientific, India) by transferring through a series of gradually increasing percentages of alcohol. The tissue samples were embedded in paraffin using embedding machine (EG1150 H&C, Leica Microsystems, Germany), sectioned using a microtome (Rotary Microtome Leica RL2125RT, Leica Microsystems, Germany) stained with hematoxylin and eosin. These samples were viewed under light microscope to observe the inflammatory responses on the region of the implant. 

The presence of neutrophils, lymphocytes, macrophages, and giant cells was used as evidence of tissue response by an independent pathologist. The inflammatory response to the implanted polymers was determined based on the average number of cell types present in the tissue surrounding implant. The average number of inflammatory cells was counted in 40x magnification from at least 10 fields, examined and tissue reaction was quantified as expressed in Table 1. Tissue responses were evaluated as minimal, mild, or moderate using an evaluation system in the literatures [7, 27, 28], and polymers were segregated as per levels 1–4 based on the biocompatibility as reported in the literatures [29–32].

### 2.5. *In Vivo* Degradation

The second implant was taken from the other side of the incision, and the tissue was slowly removed. The implant was washed and then dried under vacuum. The dried sample was then dissolved in THF and the change in the molecular weight of implant was determined using a gel permeation chromatography (GPC, Agilent 1200 Series, USA) by injecting 50 *μ*L of each sample. Molecular weight (MW) loss percentage was calculated using the following formulae

(1) MW  loss  percentage=Initial  MW  of  sample−MW  of  degraded  sampleInitial  MW  of  sample×100.

## 3. Results

### 3.1. Surface Morphology of Fabricated Implants

**Figure 1:**
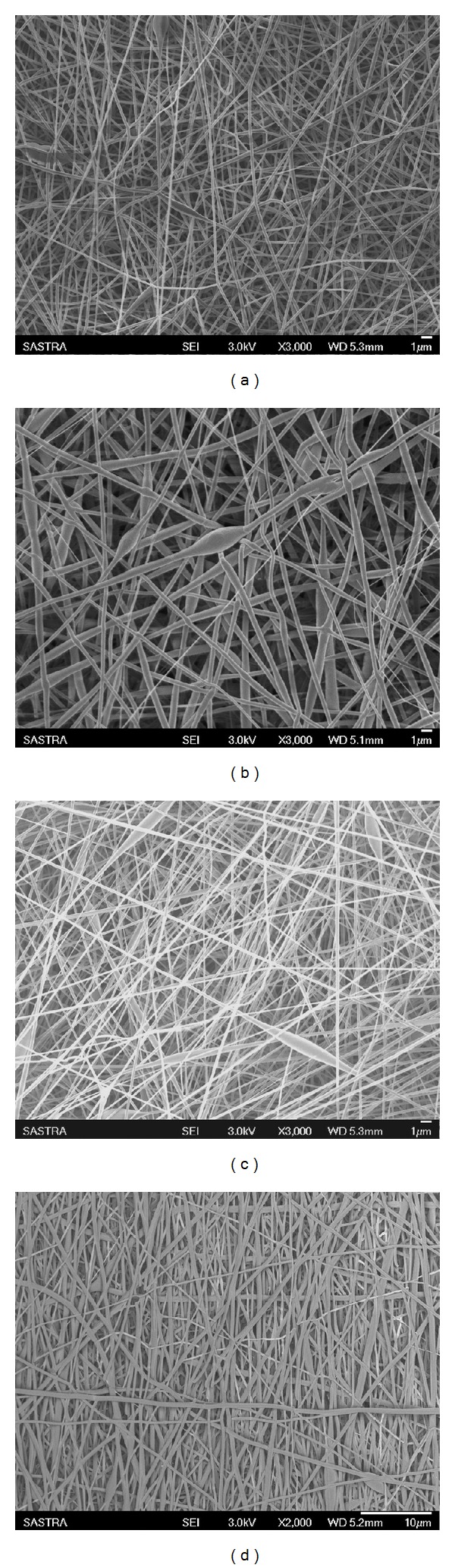
Scanning electron micrographs of (a) random PLGA, (b) random PLGA-undoped PHT, (c) random PLGA-doped PHT, and (d) aligned PLGA-doped PHT nanofibers.

In this study, we have systematically optimized the various process parameters such as flow rate, applied voltage, tip-target distance, and the solution parameters (viscosity, molecular weight, and solution conductivity) to obtain defect-free random fibers of PLGA, PLGA-undoped PHT, PLGA-doped PHT, and aligned PLGA-doped PHT nanofibers.  Figure 1 shows random and aligned nanofibers obtained at the optimized conditions shown in Table 2. The surface morphology of all implants was smooth and the diameter of random PLGA nanofibers was 197 ± 72 nm (Figure 1(a)), PLGA-undoped PHT was 201 ± 30 nm (Figure 1(b)), and PLGA-doped PHT was 196 ± 98 nm (Figure 1(c)). The diameter of aligned nanofibers of PLGA-doped PHT nanofibers was 200 ± 80 nm (Figure 1(d)).

### 3.2. Histology

**Figure 2:**
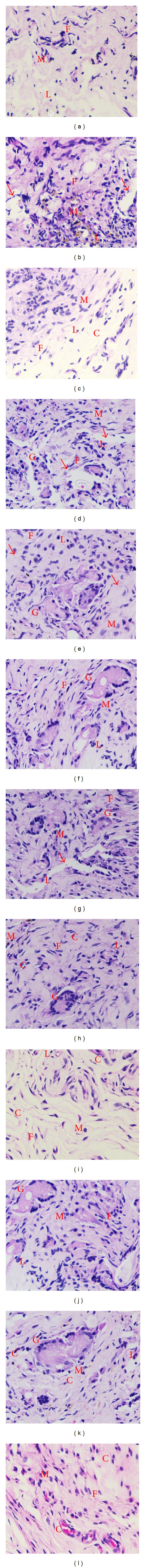
Micrographs of hematoxylin-eosin-stained tissue implanted with PLGA random nanofibers at (a) 2 weeks, (b) 4 weeks, and (c) 8 weeks; with PLGA-undoped PHT random fibers at (d) 2 weeks, (e) 4 weeks, and (f) 8 weeks; with PLGA-doped PHT random fibers at (g) 2 weeks, (h) 4 weeks, and (i) 8 weeks; and with PLGA-doped PHT aligned nanofibers at (j) 2 weeks, (k) 4 weeks, and (l) 8 weeks. C: collagen; L: lymphocytes; M: macrophages; F: fibroblast; G: giant cell; and arrows indicate implant sites.

Fibrous encapsulation was present around the implant for all groups. Further, all implants lost its structural integrity and had been disintegrated inside the fibrous capsule. Further, tissue response was quantified as in Table 1 and inflammatory responses to biomaterials at different time points (2, 4, and 8 weeks) were shown in Table 3. PLGA random fiber implanted tissue section showed a mild inflammatory response characterized by the presence of lymphocytes and negligible presence of giant cells (Figure 2(a)). Acute inflammation, abscess formation, and tissue necrosis were absent. At 4 weeks, there was a progression in the intensity of tissue reaction that was moderate with numerous pigmented/nonpigmented macrophages, lymphocytes, and frequent giant cells (Figure 2(b)). The inflammatory infiltrate had diminished at 8 weeks with few macrophages and lymphocytes (Figure 2(c)).

Random PLGA-undoped PHT nanofibrous implant established moderate tissue response characterized by the presence of predominant lymphocytes, macrophages, fibroblasts, and few giant cells (Figure 2(d)). Polymorphonuclear leukocytes were rare. The inflammatory response elicited did not reduce with time and showed moderate inflammatory response throughout the study primarily involving macrophages, lymphocytes, and giant cells (Figures 2(d)–2(f)). At 2 weeks after implantation of PLGA-doped PHT nanofibrous scaffold, fibrous tissue around the implants was observed with the complete absence of abscess formation and tissue necrosis. All implants showed a moderate tissue reaction characterized by the presence of numerous macrophages, lymphocytes, fibroblasts, frequent giant cells, and rare polymorphonuclear leukocytes (Figure 2(g)). Even at 4 weeks, there was a persistence of moderate tissue response with numerous macrophages, lymphocytes, and giant cells (Figure 2(h)). At 8 weeks, there was only mild inflammatory reaction with the decrease in the number of macrophages, lymphocytes, and giant cells (Figure 2(i)). Unlike group II, tissue responses of group III animals were diminished with time and the tissue was more organised with relative increase in the fibroblast and moderate collagen deposition.

By 2 weeks, the tissue surrounding the aligned PLGA-doped PHT nanofibers demonstrated a moderate tissue response characterised by a predominant infiltrate of lymphocytes, macrophages, and giant cells (Figure 2(j)). Acute inflammation, abscess formation, and tissue necrosis were not observed throughout the study period. After 4 weeks, the tissue bearing group IV implant evoked a decreased inflammatory response, and the inflammatory infiltrate characterised by the presence of mild lymphocytes and negligible giant cells (Figure 2(k)). The inflammatory infiltrate surrounding the aligned PLGA-doped PHT nanofibers remained the same through 8 weeks (Figure 2(l)). Like group 3, the tissue was organized well with the relative increase in the fibroblast and collagen. 

### 3.3. *In Vivo* Degradation

**Figure 3:**
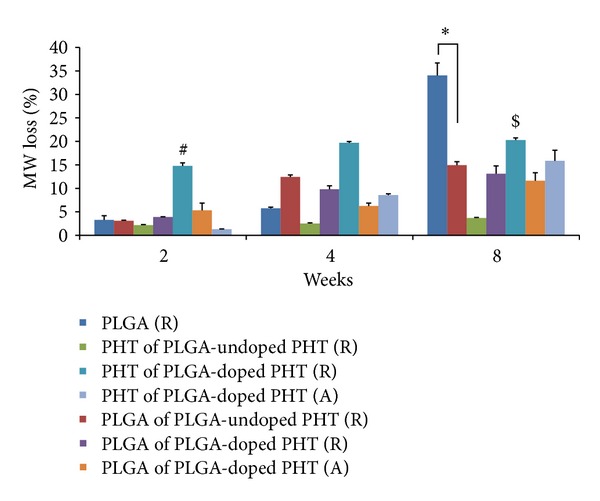
*In vivo* degradation of 2D random and 3D uniaxially aligned scaffolds as a function of molecular weight loss over time (2, 4, and 8 weeks); ∗ indicates the statistical significance with respect to PLGA of PLGA-doped PHT (A) at *P* < 0.05; #, $ indicate the statistical significance of PHT degradation in PLGA-doped PHT (R) with respect to PHT of PLGA-undoped PHT (R) and PHT of PLGA-doped PHT (A) at *P* < 0.05, respectively.

*In vivo* degradation behavior of all four implants (random PLGA, PLGA-undoped PHT, PLGA-doped PHT, and aligned PLGA-doped PHT nanofibers) were evaluated by determining the change in molecular weight of PLGA and PHT (Figure 3). Further two-way analysis of variance (ANOVA) was performed to demonstrate the effect of materials and time points on degradation rate, and post hoc Tukey test was used to analyse which means were different from others. PLGA random nanofibers showed 34.0% molecular weight loss after eight weeks. However, PLGA in PLGA-doped PHT (A) fibers demonstrated significant decline in molecular weight of 11.6% after eight weeks which was significantly lesser as compared to PLGA (R) and PLGA of PLGA-undoped PHT (R) samples at eight weeks (*P* < 0.05). For PHT degradation, undoped form of PHT was found to have significantly lesser degradation rate with respect to doped form of PHT random fibers (*P* < 0.05). Conversely, degradation rate of PHT in PLGA-doped PHT (A) was comparable to that of PHT of PLGA-undoped PHT (R) after 2 weeks (*P* > 0.05). At the end of eight weeks, undoped form of PHT did not show significant change in the molecular weight. Unlike undoped form of PHT, molecular weight of the doped PHT in random PLGA-doped PHT had reduced by 21% after eight weeks which was significantly higher with respect to PHT degradation in aligned PLGA-doped PHT (*P* < 0.05). In addition, degradation of PLGA and PHT in aligned scaffold was found to be increased gradually as compared to random scaffolds from 2 weeks to 8 weeks.

## 4. Discussion

Materials that have been used as drug delivery vehicle and tissue engineering scaffold need to be biocompatible and biodegradable [33]. Moreover, the degradation products of the material need to be nontoxic and should be eliminated from the system easily [34]. However, most of the conducting polymers are not degradable and are suspected to induce certain harmful effects in system [33]. Hence, *in vivo* biocompatibility of polymeric scaffolds evaluated using animal model, particularly in a rat model, has been a standard method to detect the tissue response [7]. Kamalesh et al. subcutaneously implanted the polyaniline films into male Sprague-Dawley rats for 90 weeks and found the noninflammatory response of the polymers throughout the study period [12]. Tissue inflammation and fibrous encapsulation are the most normal host defence responses against the foreign material [7]. In the present study, we demonstrated that the biocompatibility and biodegradability of novel blend of PLGA-PHT nanofibrous scaffold using subcutaneous rat model.

Based on the inflammatory response, the biocompatibility of the material is categorised from level 1 to level 4 [7]. Material that elicits very mild inflammatory response is termed as level 1 biocompatible material, for example, poly(hydroxyethyl methacrylate), polyhydroxyalkanoate [29, 35]. The material like zinc oxide-eugenol cements evokes mild-to-moderate response initially and the inflammatory response subsides with time, termed as level 2 biocompatible materials [30]. Materials which provoke moderate-to-severe inflammatory responses are categorized as level 3 biocompatible materials [31], and materials that elicit severe inflammatory response and do maintain the same response over time are named as level 4 biocompatible materials [32].

In this study, the PLGA random nanofibers showed a mild inflammatory response after two weeks, a moderate response after four weeks, and a mild response after eight weeks. The tissue response of PLGA random fibers diminished with time, thereby categorized as level 2 biomaterials. The molecular weight of PLGA was reduced to 34.0% after eight weeks (Figure 3), confirming the degradation behavior as well as the nontoxic nature of its degradation products. 

Our results for tissue response to random PLGA-undoped PHT nanofibers demonstrated the moderate tissue response which did not decrease over time. Hence, it was considered as level 3 biomaterial. GPC results indicated that undoped PHT did not degrade (Figure 3) and hence could influence the moderate tissue response throughout the study.

The response to random PLGA-doped PHT nanofibers was categorised as moderate after two and four weeks and then reduced to mild after eight weeks. The molecular weight of doped form of PHT was reduced as 20.5% from initial weight (Figure 3), thereby confirming its biodegradation. Moreover, the mild response after eight weeks established the noninflammatory nature of the degradation products. The addition of impurity or dopant into the electroactive polymers disturbs the rigid molecular backbone of the polymer which may improve the degradation behaviour of such material [36].

Interestingly, aligned PLGA-doped PHT nanofibers showed moderate response at two weeks, reduced to mild response at four weeks, and maintained the same response even after eight weeks. The lesser tissue response of such aligned scaffold was mainly due to the slower degradation of implant. Molecular weight loss percentage of both PHT (15.8%) and PLGA (11.6%) in aligned fiber was lesser than its respective random fibers after eight weeks of implantation (Figure 3). We have also observed slower and gradual reduction of molecular weight loss of polymeric fibers in aligned fibers than its respective random fibers. This is also one of the reasons for the mild tissue response after four and eight weeks. Though the implants in all groups show varying levels of tissue response, the results of our study classified the PLGA random, PLGA-doped PHT random, and PLGA-doped PHT aligned fibers as level 2 biocompatible materials based on the literature [27]. However, the PLGA-undoped PHT random electrospun scaffold was considered as level 3 biomaterial.

## 5. Conclusion

The main objective of the present study was to assess tissue response and degradation behaviour of novel blend of random nanofibers of PLGA-undoped PHT, PLGA-doped PHT, and aligned PLGA-doped PHT nanofibers using subcutaneous rat models. The PLGA random nanofibers were used as a control for these experiments. The results demonstrated that these polymeric nanofibers were biocompatible as well as biodegradable and can be used for tissue engineering applications. In the present study, degradation rates of different scaffolds were evaluated based on the molecular weight loss percentage. Our results also revealed that the degradation rate of aligned fibers was found to be slower and very controlled than its respective random one. Thus, the electrospun novel blend of PLGA-PHT nanofibers proved to be promising candidate for tissue engineering scaffolds because of its excellent biocompatibility and biodegradability.

## Figures and Tables

**Table 1:** Quantification of the inflammatory cells present in the fibrous tissue around the implants at different time points (2, 4, and 8 weeks).

Cell type	Cell number	Quantification
(i) Lymphocytes	0–3	+
(ii) Polymorphonuclear leucocytes (PMNs)	4–6	++
(iii) Giant cells	7–9	+++
(iv) Plasma cells	10–12	++++

**Table 2:** Optimized parameters for random and aligned nanofibers.

Polymers	Applied voltage (kV)	Flow rate (mL/min)	Needle Gauge (G)	Distance (cm)	Speed of mandrel (rpm)
PLGA (random)	25	0.001	24 G	12 cm	—
PLGA-undoped PHT (random)	25	0.004	24G	12 cm	—
PLGA-doped PHT (random)	17	0.001	22 G	12 cm	—
PLGA-doped PHT (aligned)	17	0.001	22 G	12 cm	1750

**Table 3:** Inflammatory response to random PLGA, random PLGA-undoped PHT, random PLGA-doped PHT, and aligned PLGA-doped PHT nanofibers after 2, 4, and 8 weeks of implantation. Mod: moderate.

	PLGA (R)			PLGA-undoped PHT (R)			PLGA-doped PHT (R)			PLGA-doped PHT (A)		
	2 W	4 W	8 W	2 W	4 W	8 W	2 W	4 W	8 W	2 W	4 W	8 W
Lymphocytes	++++	++++	+++	++++	+++	+++	++++	+++	+++	++++	+++	+++
PMNs	++	−	−	−	−	−	−	−	−	−	−	−
Giant cells	+	+	−	++	++	++	++	++	+	++	+	−
Plasma cells	−	−	−	−	+	+	−	−	+	−	−	−
Tissue response	Mild	Mod	Mild	Mod	Mod	Mod	Mod	Mod	Mild	Mod	Mild	Mild
