# Corrigendum to “*In Vivo* Biocompatibility of PLGA-Polyhexylthiophene Nanofiber Scaffolds in a Rat Model”

**DOI:** 10.1155/2019/2924916

**Published:** 2019-08-21

**Authors:** Anuradha Subramanian, Uma Maheswari Krishnan, Swaminathan Sethuraman

**Affiliations:** Center for Nanotechnology & Advanced Biomaterials, School of Chemical & Biotechnology, SASTRA University, Thanjavur 613 401, Tamil Nadu, India

In the article titled “*In Vivo* Biocompatibility of PLGA-Polyhexylthiophene Nanofiber Scaffolds in a Rat Model” [1], an incorrect image was provided for Figure 2(j). The corrected figure is shown below.

## Figures and Tables

**Figure 2:**
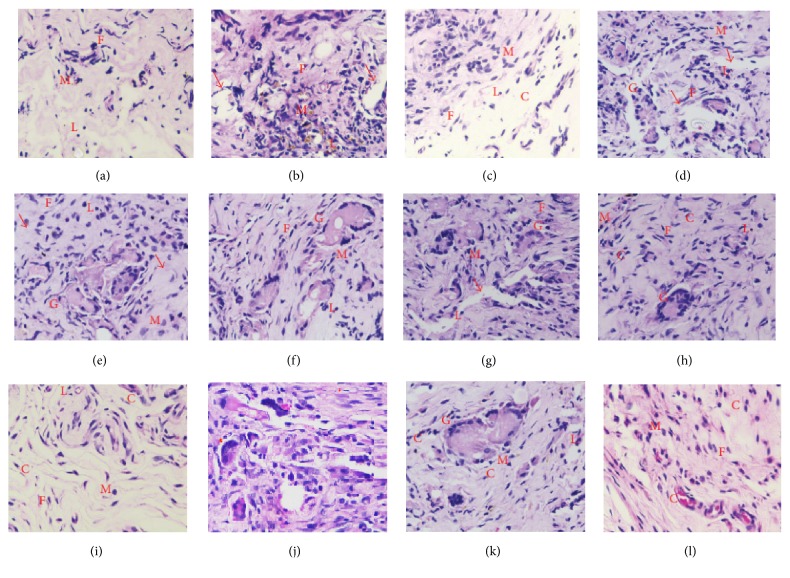
Micrographs of hematoxylin-eosin-stained tissue implanted with PLGA random nanofibers at (a) 2 weeks, (b) 4 weeks, and (c) 8 weeks; with PLGA-undoped PHT random fibers at (d) 2 weeks, (e) 4 weeks, and (f) 8 weeks; with PLGA-doped PHT random fibers at (g) 2 weeks, (h) 4 weeks, and (i) 8 weeks; and with PLGA-doped PHT aligned nanofibers at (j) 2 weeks, (k) 4 weeks, and (l) 8 weeks. C: collagen; L: lymphocytes; M: macrophages; F: fibroblast; G: giant cell; and arrows indicate implant sites.

## References

[1] Subramanian A., Krishnan U. M., Sethuraman S. (2013). In vivo biocompatibility of PLGA-polyhexylthiophene nanofiber scaffolds in a rat model. *BioMed Research International*.

